# Plasma Membrane Lipids: An Important Binding Site for All Lipoprotein Classes

**DOI:** 10.3390/membranes11110882

**Published:** 2021-11-17

**Authors:** Markus Axmann, Birgit Plochberger, Mario Mikula, Florian Weber, Witta Monika Strobl, Herbert Stangl

**Affiliations:** 1 School of Medical Engineering and Applied Social Sciences, University of Applied Sciences Upper Austria, Garnisonstrasse 21, 4020 Linz, Austria; Markus.Axmann@fh-linz.at (M.A.); Birgit.Plochberger@fh-linz.at (B.P.); Florian.Weber2@fh-linz.at (F.W.); 2 Center for Pathobiochemistry and Genetics, Institute for Medical Genetics, Medical University of Vienna, Währingerstrasse 10, 1090 Vienna, Austria; mario.mikula@meduniwien.ac.at; 3 Center for Pathobiochemistry and Genetics, Institute for Medical Chemistry, Medical University of Vienna, Währingerstrasse 10, 1090 Vienna, Austria; witta.strobl@meduniwien.ac.at

**Keywords:** non-esterified cholesterol, plasma membrane, lipoprotein particles, HDL, LDL

## Abstract

Cholesterol is one of the main constituents of plasma membranes; thus, its supply is of utmost importance. This review covers the known mechanisms of cholesterol transfer from circulating lipoprotein particles to the plasma membrane, and vice versa. To achieve homeostasis, the human body utilizes cellular de novo synthesis and extracellular transport particles for supply of cholesterol and other lipids via the blood stream. These lipoprotein particles can be classified according to their density: chylomicrons, very low, low, and high-density lipoprotein (VLDL, LDL, and HDL, respectively). They deliver and receive their lipid loads, most importantly cholesterol, to and from cells by several redundant routes. Defects in one of these pathways (e.g., due to mutations in receptors) usually are not immediately fatal. Several redundant pathways, at least temporarily, compensate for the loss of one or more of them, but the defects trigger systemic diseases, such as atherosclerosis later on. Recently, intracellular membrane–membrane contact sites were shown to be involved in intracellular cholesterol transfer and the plasma membrane itself has been proposed to act as a binding site for lipoprotein-mediated cargo unloading.

## 1. Introduction

Cholesterol is an essential constituent of cellular membranes [1]. It is not uniformly allocated among cell compartments and membranes [2], leading to a spatial and temporal concentration gradient between membrane partitions [3]. Most cholesterol in the plasma membrane is sequestered in specific areas enriched with sphingomyelins and proteins. Cholesterol exchange between the plasma membrane and intracellular compartments/membranes is of utmost importance for its distribution and for the buffering of excess cholesterol [4]. Three differently accessible cholesterol pools in the plasma membrane, with different transport rates to the endoplasmic reticulum (ER), have been identified [5,6,7].

**Figure 1:**
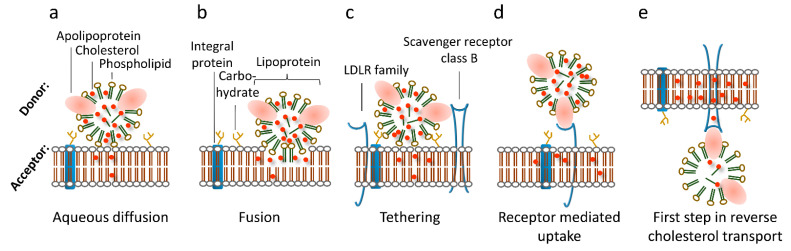
Modes of lipoprotein-mediated cargo transfer to/from cells. (**a**) Aqueous diffusion transfers cholesterol from lipoprotein particles to membranes in close proximity by passing the aqueous layer between the lipid membranes. (**b**) Fusion of lipoprotein particles yields transfer of all components of the lipoprotein particle. (**c**) Tethering function of receptors or the glycocalyx mediates aqueous diffusion. (**d**) Receptor-mediated endocytosis occurs by specific binding of certain apolipoproteins by their respective receptors and subsequent endocytosis. (**e**) Cholesterol efflux via ABC cholesterol transporters removes excess cholesterol from the cell to cholesterol-poor HDL particles.

In general, cellular cholesterol levels are controlled via de novo cholesterol synthesis as well as cholesterol uptake and release [8]. Exogenous cholesterol is mostly taken up from animal-based food. Furthermore, the majority of de novo cholesterol biosynthesis occurs in the liver [9], the brain where the blood–brain barrier impedes the exchange with cholesterol from circulation [10], and at sites of major consumption, such as the adrenals, which use cholesterol as precursor for steroid hormones [11]. As cholesterol is insoluble in water, appropriate transport vehicles, called lipoprotein particles, are required for its distribution via body fluids to facilitate transfer from the liver to the periphery and vice-versa [12]. The cholesterol exchange between cells and external sources via circulating lipoprotein particles is of central importance and cells have acquired a redundant set of different lipid transfer mechanisms to ensure proper cholesterol delivery. In addition to well characterized pathways, such as receptor-mediated endocytosis, cholesterol efflux, and selective cholesteryl ester transfer from lipoprotein particles to cells, receptor-independent transfer of non-esterified cholesterol between lipoprotein particles and the cell membrane has been described [13] (see Figure 1). In the following, we combine data that emphasize that one of these receptor independent transfer mechanisms is the interaction of lipoprotein particles with lipids of the plasma membrane itself. We show that this mechanism is functional for all lipoprotein classes, at least in artificial lipid bilayers. We argue that this route may serve to prime the plasma membrane with cholesterol, especially when protein-based pathways are impaired by mutations.

## 2. Intracellular Transfer Pathways

Like transport of cholesterol between organs and tissues of an organism, the transfer of intracellular cholesterol, originating from either de novo synthesis or uptake, is mediated by vesicular transport [14,15]. Additionally, membrane–membrane contact sites have come into the researcher’s focus [16]. Recently, evidence for direct membrane–membrane cholesterol transport between the plasma membrane and the ER was presented by Peter Tontonoz’s group [17]. There, non-vesicular transport of high-density lipoprotein (HDL)-derived cholesteryl moieties hydrolyzed from cholesteryl esters (CEs) was described to occur via the Aster (GramD) protein family directly from the plasma membrane to the ER [17]. Furthermore, Aster proteins control the accessible cholesterol pools in the plasma membrane and ER [18], demonstrated by the activity of the sterol regulatory element-binding protein (SREBP), a protein that senses the ER cholesterol content [19]. Similarly, Bruno Antonny’s group reported sterol transfer between ER and the trans-Golgi network [20]. Thus, intracellular cholesterol transport can be facilitated by direct membrane–membrane contact [21], where Aster proteins form bridges between the ER and plasma membrane. One may envision that the transfer of non-esterified cholesterol from lipoprotein particles, which contains phospholipid shell-like intracellular membranes, to the plasma membrane may follow a similar (if not the same) mechanism involving a highly accessible pool of plasma membrane cholesterol. 

## 3. Receptor-Mediated Cholesterol Uptake, Efflux, and Transcytosis Pathways

The main delivery pathway for low-density lipoprotein (LDL)-derived cholesterol (LDL-C) is its uptake via clathrin-coated pits by endocytosis of LDL particles, the dominant carrier of circulating cholesterol. The majority of LDL receptors are located on the surface of hepatocytes, although cells of virtually all other tissues express the LDL receptor [22,23,24]. Usually, they are located in coated pits on the surface of cells, and bind LDL particles specifically (with high affinity). After endocytosis, the LDL particle is released from its receptor in a pH and calcium dependent manner within early endosomes [24]. Degradation of LDL particles, including hydrolysis of its CEs by acid lipase, is proposed to take place during or after the structural and functional transition of early into late endosomes. The receptor recycles back to the plasma membrane and is ready to participate in a new round of LDL particle endocytosis. This holo particle uptake is called “LDL receptor-mediated endocytosis” [23]. In addition to lipoprotein particles carrying the apolipoprotein ApoB, proteins of the LDL receptor family bind ApoE containing lipoprotein particles. In this context, heparan sulfate proteoglycans (HSPGs) enhance the uptake of lipoprotein particles and mediate their catabolism [25]. In line, HSPGs are involved in the uptake of remnant lipoprotein particles—a smaller chylomicron particle—by the LDL receptor-related protein (LRP) [26,27,28,29]. HSPGs may act as anchors for tethering remnant particles and other lipoprotein particles in close proximity to the cell membrane and thereby mediate cholesterol transfer (see Figure 1). This process may occur mainly in capillaries where the blood flow is low.

In humans, HDL-derived lipids are removed from the circulation by at least two pathways: an indirect pathway in which CEs from HDL particles are transferred to LDL or VLDL particles facilitated by cholesteryl ester transfer protein (CETP) [30], and a direct one via selective lipid uptake by the liver [31], mediated by the selective cholesteryl ester uptake pathway via an integral cell membrane protein named scavenger receptor class B type 1 (SR-B1) [32,33,34]. This was demonstrated by the transfer of radiolabeled CEs and the neutral phospholipid 1,1′-dioctadecyl-3,3,3′,3′-tetramethylindocarbocyanine perchlorate (DiI) [33,35,36]. SR-B1 binds HDL particles and mediates transfer of cargo lipids from the particle to the ER via the above-mentioned mechanism involving Aster-B [17]. Non-esterified cholesterol from HDL particles is considered to enter cells after desorption from the particles by diffusion through the aqueous environment into the cell membrane [37,38,39] (see Figure 1).

Other cell membrane proteins, such as the ATP-binding cassette transporter subfamily A member 1 (ABCA1), act in the opposite direction and are essential for cellular cholesterol efflux, which is the first step in reverse cholesterol transport (RCT, see Figure 1) [40]. In addition to ABCA1, SR-B1 facilitates cholesterol efflux to mature HDL particles, most likely via passive diffusion [32]. At least two lipoprotein particle-binding sites have been described for SR-B1: a high-affinity site recognizing multiple α-helical sites on ApoA1 [41], and a second site with a lower affinity recognizing specific lipids at the particle’s surface [42]. RCT operates not exclusively on ApoA1 containing lipoprotein particles—the main protein constituent of HDL particles—but also on ApoB containing (thus larger) ones like LDL particles. Overall, RCT is a highly regulated process that relies on specific interactions between HDL particles and cell membranes. It involves multiple steps and components, such as cholesterol efflux from lipid-laden macrophages, HDL-modifying enzymes, and hepatic HDL receptors [32]. The nature of the subsequent cargo transfer process is still a matter of discussion: it occurs either at the cell surface [37,43] or after holo HDL particle endocytosis [44,45,46,47]. One hypothesis states that relatively fluid and disordered membrane domains facilitate trafficking of cholesterol between HDL particles and the target membrane with a subsequent redistribution to other membrane domains [48]. Consistently, SR-B1 translocates from disordered to ordered membrane domains upon external stimuli [49,50] and plasma membrane cholesterol levels influence selective CE uptake via SR-B1 [51,52]. It is conceivable that the second “lower affinity binding site”, described by Monty Krieger’s group, is the plasma membrane itself. In support of this concept, we have shown that the cholesterol transfer rate to cholesterol-starved cells mainly depends on the cholesterol content of the lipoprotein particle in cell lines overexpressing SR-B1 [53,54]. The cholesterol transfer rates of LDL and HDL particles to cells were similar after correcting for their different cholesterol contents [53,54]. If in tissue culture experiments the ratio between lipoprotein particles and cells is kept constant, the transport direction of the tracer ^3^H-cholesterol depends only on its location. Thus, the tracer moves always along the preexisting gradient between lipoprotein particles and the cell [54]. Together, these results demonstrate that non-esterified cholesterol is transferred along a concentration gradient independent of the lipoprotein particle’s type. In addition to LDL and HDL particles, SR-B1 also mediates transfer of very low-density lipoprotein (VLDL)-derived lipids, [55], which underlines the functionality of the second lipid-binding site described by Monty Krieger (see above and [42]). It has to be noted that holo HDL particle uptake is observed in tissue culture cells, which are often of cancer origin. In the corresponding tissues, this pathway is much less expressed (Röhrl, Kovacs, Stangl, unpublished data). As many cancer cells have activated uptake pathways to meet their increased demand for building blocks [56], they may also have altered membrane composition, which may result in higher lipoprotein particle uptake/transcytosis. For example, it has been shown that ovarian tumor cells scavenge cholesterol from tumor-associated macrophages, resulting in a tumor-promoting phenotype [57].

Lipoprotein particle receptors have also been implicated in particle transcytosis. The research group of von Eckardstein extensively characterized lipoprotein particle transcytosis in an endothelial trans-well model [58]. Several receptors are involved in transcytosis of LDL and HDL particles: SR-B1 mediates HDL as well as LDL particle transcytosis. Furthermore, ATP-binding cassette transporter Subfamily G Member 1 (ABCG1) and endothelial lipase are involved in HDL transcytosis; LDL transcytosis is influenced by activin like kinase 1, and caveolin-1 [58]. Inactivation of cell surface receptors as discussed in the next chapter, even double knock-down of SR-B1 and ABCG1, did not shutdown lipoprotein particle transcytosis [59]. It is therefore conceivable that still unknown mechanisms mediate uptake of lipoprotein particles in this context. Additionally, the lipids of the plasma membrane itself may act as a binding site contributing to transcytosis and possibly functioning as a sort of overflow sink for excess lipoprotein particles.

## 4. Redundancy of Cholesterol Transfer Pathways

The redundancy of cholesterol transfer pathways in combination with de novo cholesterol biosynthesis explains the observation that genetic disorders of lipoprotein metabolism in general do not lead to intrauterine death. Ultimately, over a longer period of time, all mutations discussed below will lead to atherosclerosis and cardiovascular diseases. However, their relatively late onset proves the existence of robust and redundant alternative pathways of cellular cholesterol supply and distribution. In this context, de novo cholesterol biosynthesis can compensate for the lack of exogenous supply. Endogenous cholesterol biosynthesis, however, is not homogenous within the body and thus cholesterol distribution within the organism and especially RCT are of great importance for cholesterol homeostasis.

Familial hypercholesterolemia (FH) occurs due to an altered pathway of receptor-mediated LDL uptake due to mutations of the LDL receptor, the ApoB protein, the low-density lipoprotein receptor adapter protein 1 (LDLRAP1), or the proprotein convertase subtilisin/kexin type 9 (PCSK9) gene [60]. Mutations in the ARH gene lead to familial combined hypercholesterolemia, which also limits receptor-mediated endocytosis by a different mechanism [61]. Mutations in the SR-B1 [62] gene, but also in ABC cholesterol transporter gene do not result in premature death or a very severe phenotype—ABCA1 and ABCG5/8 gene mutations lead to Tangier disease [63] and sitosterolemia [64], respectively. None of these mutations causes miscarriage, but the high total and LDL-C levels in the blood stream will lead to an early onset of atherosclerosis.

Similar results can be observed in several mammalian species: dogs with a mutation in the CommD1 gene, which inhibits receptor-mediated endocytosis/receptor recycling, are vital [65]. Rabbits harboring a mutation in the LDL receptor survive to adulthood and are used as a model for FH [66]. In line, genetic model rodents survive with an inactivation of the LDL receptor-mediated cholesterol uptake pathway by harboring a mutation in the LDL-receptor gene or the ApoE gene, which codes for the major apolipoprotein in mice and other genes associated with the LDL-receptor family, such as VLDLR or ApoE4 receptor without severe atherosclerosis [67]. It should be noted that mice in general have low LDL-C levels and higher HDL-C levels [67] and atherosclerosis can only be triggered in knockout animals on a fat- and cholesterol-enriched westernized diet [68]. Double knockout of VLDLR and ApoE4R genes show a developmental brain defect, as many of the genes of the LDL receptor family are important for brain development [69]. Only animals with an inactivation of the LDL receptor-related protein 1 (LRP1) genes do not live to adulthood. Even triple knockout mice with an inactivation of LDL receptor and VLDL receptor and an inactivation of LRP in the liver are viable [70]; their hepatic lipid uptake is secured by HSPGs, SR-B1 and lipoprotein lipase [29]. In line, also ApoA1-, ApoA2-, and ApoE-deficient mice are viable [71]. Similar to humans, also mice with an inactivation of other cholesterol transporters like SR-B1 [72], ABCA1 [73], ABCG1 [74], or ABCG5/8 [75] show no severe phenotype. Animals with double knockout of the SR-B1 and ApoE gene are viable. However, they exhibit an early onset of occlusive coronary artery disease, spontaneous myocardial infarctions, and premature death [76]. This evidence highlights the redundancy of cholesterol supply chains and points to the direction of other (although not well characterized) uptake routes.

## 5. The Role of the Plasma Membrane Lipids for Cholesterol Uptake

Membranes are highly dynamic and can change rapidly in composition and, thus, curvature [77], which alters fusion behavior [78] to meet, among other aspects, transport requirements [3]. Membrane–membrane interactions, including particle fusion and cargo transfer [79], are regularly observed and modeled [80]. The lipid composition of membranes determines its rigidity and charge [81,82,83]. Accordingly, it is well established that the size of HDL particles define their lipid loading and unloading processes. Small lipid-poor pre-β HDL particles accept cholesterol from peripheral cells whereas they mature to larger HDL3 particles, which deliver their lipid load to the liver or steroidogenic cells.

**Figure 2:**
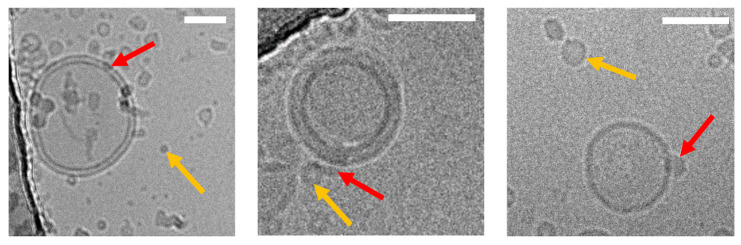
Cryo-EM images of lipoprotein particles fused to an artificial membrane mimicking the plasma membrane (unpublished data, J. Novacek). (**Left** to **right**): images of HDL, LDL, and VLDL particles fused (red arrow) to individual large unilamellar vesicles (LUV) made from 1,2-dioleoyl-sn-glycero-3-phosphocholine (DOPC). Non-fused lipoprotein particles are exemplarily indicated by the yellow arrow. Lipoprotein particle incorporation into the LUV membranes was confirmed through recording data under different electron-beam incident angles (0°, 30°, 60°, not shown), thus excluding an accidental overlay of signals originating from different layers of the vitrified ice. Images were acquired under low-dose conditions (20 e/Å^2^). Scale bars are 50 nm.

**Figure 3:**
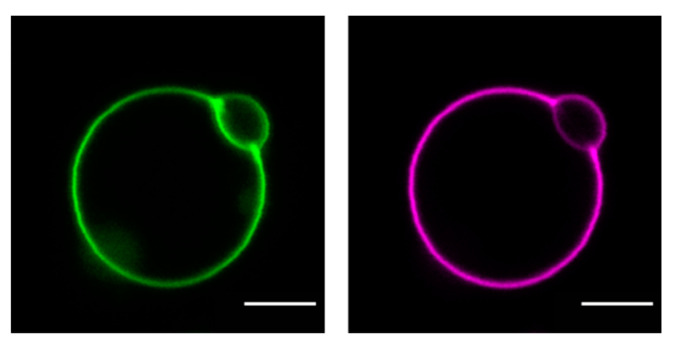
Confocal images of cholesterol and protein distribution in a GUV incubated with HDL (unpublished data, E. Sezgin): GUVs prepared from 1-palmitoyl-2-oleoyl- glycero-3-phosphocholine (POPC) were incubated with 0.05 mg/mL dual fluorescently labeled HDL particles (Cholesterol-BodipyFL (green), HDL-associated proteins-Atto647N (magenta)). Scale bar: 5 µm.

Recently, we have shown direct cholesterol transfer from HDL and LDL particles to supported lipid bilayers (SLBs). We have studied interaction and cargo transfer of HDL, LDL, and VLDL particles with SLBs and large/giant unilamellar vesicles (LUVs, GUVs) employing cryoelectron (see Figure 2) and atomic force microscopy, confocal imaging (see Figure 3), and fluorescence (cross) correlation spectroscopy (FCCS) [84,85,86]. We detected interaction forces resembling those observed during lipid tube formation between lipoprotein particles and SLBs. After incubation with fluorescently labeled lipoprotein particles, we measured the fluorescence signal of labeled cholesterol and lipoprotein-derived proteins in SLBs and GUVs and found that the diffusion of the protein fraction within the membrane was different from the situation in solutions where cholesterol and protein move together on the same particle. In particular, the FCCS data showed that after the interaction of the lipoprotein particles with the target membrane an independent diffusion of Cholesterol-BodipyFL and lipoprotein particle-associated proteins occurred [84]. This observation proves that lipoprotein particles can release their cholesterol directly at least to artificial membranes. Using an environmentally sensitive probe called NR12S, we showed that cholesterol and saturated lipids derived from lipoprotein particles rigidify the target membrane [85]. Similar results, such as a lower exchange rate for HDL and LDL particles, were recently obtained using neutron reflectometry on model membranes in the presence of cholesterol by Cárdenas’s research group [87,88].

Thus, alterations of membrane elasticity, as also seen in (patho)physiological states, might affect HDL-mediated cargo transfer. In fact, a variety of alterations in membrane composition due to specific diets or in metabolic diseases have been described, e.g., free fatty acids, such as docosahexaenoic acid (DHA), alter the elastic properties and curvatures of membranes [89]. Kahn et al. showed that 7-keto cholesterol as well as C24:0 and C26:0 fatty acid—metabolites detected in multiple sclerosis and X-linked adrenoleukodystrophy, respectively—trigger lateral lipid membrane disorganization of oligodendrocytes [90].

Besides the well-characterized cholesterol uptake pathways mentioned above, the lipids of the plasma membrane may play an important role in cholesterol transfer to and from lipoprotein particles. In this context, the plasma membrane may ultimately act as lipid-mediated non-protein-based binding site for lipoprotein particles promoting binding and fusion with the plasma membrane. In this process, cholesterol transfer is triggered by a pre-existing concentration gradient. In summary, the available evidence suggests that lipoprotein particles fuse with artificial target membranes upon membrane interaction, transfer their cargo, and, thereby, alter the biophysical properties, such as the stiffness of the target membrane. We hypothesize that such a receptor-independent process driven by a pre-existing concentration gradient is immediate in comparison to any receptor-driven one—it does not involve complicated receptor coupling and endocytic uptake. Receptor-independent delivery to the plasma membrane may supply the basal level of cellular cholesterol cargo quickly. Indeed, direct transfer of cholesterol from lipoprotein particles to the liver’s biliary side has been described previously [91,92,93,94,95].

## 6. Conclusions

In this paper, we reviewed the currently known transfer mechanisms of cholesterol, to and from the plasma membrane, and summarized our own observations that membrane lipids may act as a binding site for lipoprotein particles. Our in vitro data using protein-free artificial membranes show that cholesterol transfer can occur in the absence of any receptors. Receptors, such as SR-B1 or HSPGs, may capture lipoprotein particles from the bloodstream, tether them to the plasma membrane, and increase the likelihood of fusion. In addition, conformational fluctuations of the membrane may bring the particle closer to the membrane. When the local membrane environment is poor in cholesterol, fusion and transfer may occur. In case the cholesterol gradient is not sufficient, the particle may be repelled and dissociates. Based on these findings, we argue that such protein-independent pathways may also exist in vivo, in parallel to the well-established protein-mediated cholesterol transport routes mentioned above. Its quantitative contribution is still unknown. However, the transfer of cholesterol from lipoprotein particles directly to cellular membranes may represent an important pathway in addition to de novo cholesterol biosynthesis, to ensure cholesterol homeostasis and cellular integrity—especially in the context of impaired protein-based pathways.

## Data Availability

Raw data were generated at the Cryo-Electron Microscopy and Tomography Core Facility CEITEC MU of CIISB (*J. Novacek*) and the University of Oxford (*E. Sezgin*). The data presented in this study are available upon request from the corresponding author. The data are not publicly available due to restrictions of the involved scientists and facilities.

## References

[1] Maxfield F.R., van Meer G. (2010). Cholesterol, the central lipid of mammalian cells. Curr. Opin. Cell Biol..

[2] van Meer G. (1989). Lipid Traffic in Animal Cells. Annu. Rev. Cell Biol..

[3] Pinkwart K., Schneider F., Lukoseviciute M., Sauka-Spengler T., Lyman E., Eggeling C., Sezgin E. (2019). Nanoscale dynamics of cholesterol in the cell membrane. J. Biol. Chem..

[4] Ikonen E., Zhou X. (2021). Cholesterol transport between cellular membranes: A balancing act between interconnected lipid fluxes. Dev. Cell.

[5] Das A., Brown M.S., Anderson D.D., Goldstein J.L., Radhakrishnan A. (2014). Three pools of plasma membrane cholesterol and their relation to cholesterol homeostasis. eLife.

[6] Infante R.E., Radhakrishnan A. (2017). Continuous transport of a small fraction of plasma membrane cholesterol to endoplasmic reticulum regulates total cellular cholesterol. eLife.

[7] Johnson K.A., Radhakrishnan A. (2020). Accessibility of cholesterol at cell surfaces. J. Lipid Res..

[8] Luo J., Yang H., Song B.-L. (2020). Mechanisms and regulation of cholesterol homeostasis. Nat. Rev. Mol. Cell Biol..

[9] Brown M.S., Goldstein J.L., Siperstein M.D. (1973). Regulation of cholesterol synthesis in normal and malignant tissue. Fed. Proc..

[10] Björkhem I., Meaney S. (2004). Brain Cholesterol: Long Secret Life Behind a Barrier. Arter. Thromb. Vasc. Biol..

[11] Gwynne J.T., Strauss J.F. (1982). The Role of Lipoproteins in Steroidogenesis and Cholesterol Metabolism in Steroidogenic Glands. Endocr. Rev..

[12] Ehuang L.-H., Eelvington A., Randolph G.J. (2015). The role of the lymphatic system in cholesterol transport. Front. Pharmacol..

[13] Axmann M., Strobl W.M., Plochberger B., Stangl H. (2019). Cholesterol transfer at the plasma membrane. Atherosclerosis.

[14] Ikonen E. (2008). Cellular cholesterol trafficking and compartmentalization. Nat. Rev. Mol. Cell Biol..

[15] Ikonen E., Kanerva K. (2019). Shuttling HDL Cholesterol to the Membrane via Metastable Receptor Multimers. Dev. Cell.

[16] Prinz W.A., Toulmay A., Balla T. (2020). The functional universe of membrane contact sites. Nat. Rev. Mol. Cell Biol..

[17] Sandhu J., Li S., Fairall L., Pfisterer S.G., Gurnett J.E., Xiao X., Weston T.A., Vashi D., Ferrari A., Orozco J.L. (2018). Aster Proteins Facilitate Nonvesicular Plasma Membrane to ER Cholesterol Transport in Mammalian Cells. Cell.

[18] Ferrari A., He C., Kennelly J.P., Sandhu J., Xiao X., Chi X., Jiang H., Young S.G., Tontonoz P. (2020). Aster Proteins Regulate the Accessible Cholesterol Pool in the Plasma Membrane. Mol. Cell. Biol..

[19] Radhakrishnan A., Goldstein J.L., McDonald J.G., Brown M.S. (2008). Switch-like Control of SREBP-2 Transport Triggered by Small Changes in ER Cholesterol: A Delicate Balance. Cell Metab..

[20] Mesmin B., Bigay J., Polidori J., Jamecna D., Lacas-Gervais S., Antonny B. (2017). Sterol transfer, PI 4P consumption, and control of membrane lipid order by endogenous OSBP. EMBO J..

[21] Naito T., Saheki Y. (2021). GRAMD1-mediated accessible cholesterol sensing and transport. Biochim. Biophys. Acta Mol. Cell Biol. Lipids.

[22] Brown M.S., Goldstein J.L. (1986). A receptor-mediated pathway for cholesterol homeostasis. Science.

[23] Goldstein J.L., Brown M.S., Anderson R.G.W., Russell D., Schneider W.J. (1985). Receptor-Mediated Endocytosis: Concepts Emerging from the LDL Receptor System. Annu. Rev. Cell Biol..

[24] Goldstein J.L., Brown M.S. (2009). The LDL Receptor. Arter. Thromb. Vasc. Biol..

[25] Mahley R.W., Ji Z.S. (1999). Remnant lipoprotein metabolism: Key pathways involving cell-surface heparan sulfate proteo-glycans and apolipoprotein E. J. Lipid Res..

[26] Ji Z., Brecht W., Miranda R., Hussain M., Innerarity T., Mahley R. (1993). Role of heparan sulfate proteoglycans in the binding and uptake of apolipoprotein E-enriched remnant lipoproteins by cultured cells. J. Biol. Chem..

[27] Ji Z., Fazio S., Lee Y., Mahley R. (1994). Secretion-capture role for apolipoprotein E in remnant lipoprotein metabolism involving cell surface heparan sulfate proteoglycans. J. Biol. Chem..

[28] Ji Z., Fazio S., Mahley R. (1994). Variable heparan sulfate proteoglycan binding of apolipoprotein E variants may modulate the expression of type III hyperlipoproteinemia. J. Biol. Chem..

[29] Hu L., van der Hoogt C.C., Espirito Santo S.M., Out R., Kypreos K., van Vlijmen B.J.M., Van Berkel T.J.C., Romijn J.A., Havekes L.M., van Dijk K.W. (2008). The hepatic uptake of VLDL in lrpldlrvldlr mice is regulated by LPL activity and involves proteoglycans and SR-BI. J. Lipid Res..

[30] Feingold K.R., Feingold K.R., Anawalt B., Boyce A., Chrousos G., de Herder W.W., Dhatariya K., Dungan K., Grossman A., Hershman J.M., Hofland J. (2000). Introduction to Lipids and Lipoproteins. Endotext.

[31] von Eckardstein A. (2012). Tachometer for Reverse Cholesterol Transport?. J. Am. Hear. Assoc..

[32] Shen W.-J., Azhar S., Kraemer F. (2018). SR-B1: A Unique Multifunctional Receptor for Cholesterol Influx and Efflux. Annu. Rev. Physiol..

[33] Acton S., Rigotti A., Landschulz K.T., Xu S., Hobbs H.H., Krieger M. (1996). Identification of Scavenger Receptor SR-BI as a High Density Lipoprotein Receptor. Science.

[34] Stangl H., Strobl W., Komoda T. (2017). Role of SR-BI in HDL Metabolism. The HDL Handbook.

[35] Landschulz K.T., Pathak R.K., Rigotti A., Krieger M., Hobbs H.H. (1996). Regulation of scavenger receptor, class B, type I, a high density lipoprotein receptor, in liver and steroidogenic tissues of the rat. J. Clin. Investig..

[36] Gu X., Trigatti B.L., Xu S., Acton S., Babitt J., Krieger M. (1998). The Efficient Cellular Uptake of High Density Lipoprotein Lipids via Scavenger Receptor Class B Type I Requires Not Only Receptor-mediated Surface Binding but Also Receptor-specific Lipid Transfer Mediated by Its Extracellular Domain. J. Biol. Chem..

[37] Rothblat G., Mahlberg F., Johnson W., Phillips M. (1992). Apolipoproteins, membrane cholesterol domains, and the regulation of cholesterol efflux. J. Lipid Res..

[38] Phillips M.C., Johnson W.J., Rothblat G.H. (1987). Mechanisms and consequences of cellular cholesterol exchange and transfer. Biochim. Biophys. Acta.

[39] Scobey M.W., Johnson F.L., Rudel L.L. (1989). Delivery of high-density lipoprotein free and esterified cholesterol to bile by the perfused monkey liver. Am. J. Physiol. Liver Physiol..

[40] Phillips M.C. (2018). Is ABCA1 a lipid transfer protein?. J. Lipid Res..

[41] Williams D.L., de la Llera-Moya M., Thuahnai S.T., Lund-Katz S., Connelly M.A., Azhar S., Anantharamaiah G., Phillips M.C. (2000). Binding and Cross-linking Studies Show That Scavenger Receptor BI Interacts with Multiple Sites in Apolipoprotein A-I and Identify the Class A Amphipathic α-Helix as a Recognition Motif. J. Biol. Chem..

[42] Gu X., Lawrence R., Krieger M. (2000). Dissociation of the high density lipoprotein and low density lipoprotein binding activities of murine scavenger receptor class B type I (mSR-BI) using retrovirus library-based activity dissection. J. Biol. Chem..

[43] Rodrigueza W.V., Thuahnai S.T., Temel R.E., Lund-Katz S., Phillips M.C., Williams D.L. (1999). Mechanism of scavenger receptor class B type I-mediated selective uptake of cholesteryl esters from high density lipoprotein to adrenal cells. J. Biol. Chem..

[44] Silver D.L., Wang N., Xiao X., Tall A.R. (2001). High Density Lipoprotein (HDL) Particle Uptake Mediated by Scavenger Receptor Class B Type 1 Results in Selective Sorting of HDL Cholesterol from Protein and Polarized Cholesterol Secretion. J. Biol. Chem..

[45] Pagler T.A., Rhode S., Neuhofer A., Laggner H., Strobl W., Hinterndorfer C., Volf I., Pavelka M., Eckhardt E.R., van der Westhuyzen D.R. (2006). SR-BI-mediated High Density Lipoprotein (HDL) Endocytosis Leads to HDL Resecretion Facilitating Cholesterol Efflux. J. Biol. Chem..

[46] Röhrl C., Meisslitzer-Ruppitsch C., Bittman R., Li Z., Pabst G., Prassl R., Strobl W., Neumüller J., Ellinger A., Pavelka M. (2012). Combined light and electron microscopy using diaminobenzidine photooxidation to monitor trafficking of lipids derived from lipoprotein particles. Curr. Pharm. Biotechnol..

[47] Röhrl C., Pagler T.A., Strobl W., Ellinger A., Neumüller J., Pavelka M., Stangl H., Meisslitzer-Ruppitsch C. (2009). Characterization of endocytic compartments after holo-high density lipoprotein particle uptake in HepG2 cells. Histochem. Cell Biol..

[48] Peng Y., Akmentin W., Connelly M.A., Lund-Katz S., Phillips M.C., Williams D.L. (2004). Scavenger Receptor BI (SR-BI) Clustered on Microvillar Extensions Suggests that This Plasma Membrane Domain Is a Way Station for Cholesterol Trafficking between Cells and High-Density Lipoprotein. Mol. Biol. Cell.

[49] Béaslas O., Cueille C., Delers F., Chateau D., Chambaz J., Rousset M., Carrière V. (2009). Sensing of Dietary Lipids by Enterocytes: A New Role for SR-BI/CLA-1. PLoS ONE.

[50] Saddar S., Carrière V., Lee W.-R., Tanigaki K., Yuhanna I.S., Parathath S., Morel E., Warrier M., Sawyer J.K., Gerard R.D. (2013). Scavenger Receptor Class B Type I Is a Plasma Membrane Cholesterol Sensor. Circ. Res..

[51] Rhainds D., Bourgeois P., Bourret G., Huard K., Falstrault L., Brissette L. (2004). Localization and regulation of SR-BI in membrane rafts of HepG2 cells. J. Cell Sci..

[52] Rhainds D., Brissette L. (2004). The role of scavenger receptor class B type I (SR-BI) in lipid trafficking: Defining the rules for lipid traders. Int. J. Biochem. Cell Biol..

[53] Stangl H., Cao G., Wyne K.L., Hobbs H.H. (1998). Scavenger Receptor, Class B, Type I-dependent Stimulation of Cholesterol Esterification by High Density Lipoproteins, Low Density Lipoproteins, and Nonlipoprotein Cholesterol. J. Biol. Chem..

[54] Stangl H., Hyatt M., Hobbs H.H. (1999). Transport of Lipids from High and Low Density Lipoproteins via Scavenger Receptor-BI. J. Biol. Chem..

[55] Röhrl C., Fruhwürth S., Schreier S.M., Lohninger A., Dolischka A., Hüttinger M., Zemann N., Hermann M., Strobl W., Stangl H. (2010). Scavenger receptor, Class B, Type I provides an alternative means for β-VLDL uptake independent of the LDL receptor in tissue culture. Biochim. Biophys. Acta.

[56] Romero-Garcia S., Lopez-Gonzalez J.S., Báez-Viveros J.L., Aguilar-Cazares D., Prado-Garcia H. (2011). Tumor cell metabolism: An integral view. Cancer Biol. Ther..

[57] Goossens P., Rodriguez-Vita J., Etzerodt A., Masse M., Rastoin O., Gouirand V., Ulas T., Papantonopoulou O., Van Eck M., Auphan-Anezin N. (2019). Membrane Cholesterol Efflux Drives Tumor-Associated Macrophage Reprogramming and Tumor Progression. Cell Metab..

[58] Jang E., Robert J., Rohrer L., von Eckardstein A., Lee W.L. (2020). Transendothelial transport of lipoproteins. Atherosclerosis.

[59] Rohrer L., Ohnsorg P.M., Lehner M., Landolt F., Rinninger F., von Eckardstein A. (2009). High-Density Lipoprotein Transport Through Aortic Endothelial Cells Involves Scavenger Receptor BI and ATP-Binding Cassette Transporter G1. Circ. Res..

[60] Soutar A.K., Naoumova R.P. (2007). Mechanisms of Disease: Genetic causes of familial hypercholesterolemia. Nat. Clin. Pract. Cardiovasc. Med..

[61] Garcia C.K., Wilund K., Arca M., Zuliani G., Fellin R., Maioli M., Calandra S., Bertolini S., Cossu F., Grishin N. (2001). Autosomal Recessive Hypercholesterolemia Caused by Mutations in a Putative LDL Receptor Adaptor Protein. Science.

[62] Vergeer M., Korporaal S.J., Franssen R., Meurs I., Out R., Hovingh G.K., Hoekstra M., Sierts J.A., Dallinga-Thie G.M., Motazacker M.M. (2011). Genetic Variant of the Scavenger Receptor BI in Humans. N. Engl. J. Med..

[63] Bodzioch M., Orsó E., Klucken J., Langmann T., Böttcher A., Diederich W., Drobnik W., Barlage S., Büchler C., Porsch-Özcürümez M. (1999). The gene encoding ATP-binding cassette transporter 1 is mutated in Tangier disease. Nat. Genet..

[64] Berge K.E., Tian H., Graf G.A., Yu L., Grishin N.V., Schultz J., Kwiterovich P., Shan B., Barnes R., Hobbs H.H. (2000). Accumulation of Dietary Cholesterol in Sitosterolemia Caused by Mutations in Adjacent ABC Transporters. Science.

[65] Bartuzi P., Billadeau D.D., Favier R., Rong S., Dekker D., Fedoseienko A., Fieten H., Wijers M., Levels J.H., Huijkman N. (2016). CCC- and WASH-mediated endosomal sorting of LDLR is required for normal clearance of circulating LDL. Nat. Commun..

[66] Havel R.J., Yamada N., Shames D.M. (1989). Watanabe heritable hyperlipidemic rabbit. Animal model for familial hypercholesterolemia. Arteriosclerosis.

[67] Hofker M.H., van Vlijmen B.J., Havekes L.M. (1998). Transgenic mouse models to study the role of APOE in hyperlipidemia and atherosclerosis. Arteriosclerosis.

[68] Getz G.S., Reardon C.A. (2006). Diet and Murine Atherosclerosis. Arterioscler. Thromb. Vasc. Biol..

[69] Trommsdorff M., Gotthardt M., Hiesberger T., Shelton J., Stockinger W., Nimpf J., E Hammer R., A Richardson J., Herz J. (1999). Reeler/Disabled-like Disruption of Neuronal Migration in Knockout Mice Lacking the VLDL Receptor and ApoE Receptor 2. Cell.

[70] Santo S.M.S.E., Rensen P.C.N., Goudriaan J.R., Bensadoun A., Bovenschen N., Voshol P.J., Havekes L.M., van Vlijmen B.J.M. (2005). Triglyceride-rich lipoprotein metabolism in unique VLDL receptor, LDL receptor, and LRP triple-deficient mice. J. Lipid Res..

[71] Plump A.S., Erickson S.K., Weng W., Partin J.S., Breslow J.L., Williams D.L. (1996). Apolipoprotein A-I is required for cholesteryl ester accumulation in steroidogenic cells and for normal adrenal steroid production. J. Clin. Investig..

[72] Rigotti A., Trigatti B.L., Penman M., Rayburn H., Herz J., Krieger M. (1997). A targeted mutation in the murine gene encoding the high density lipoprotein (HDL) receptor scavenger receptor class B type I reveals its key role in HDL metabolism. Proc. Natl. Acad. Sci. USA.

[73] Orsó E., Broccardo C., Kaminski W.E., Böttcher A., Liebisch G., Drobnik W., Götz A., Chambenoit O., Diederich W., Langmann T. (2000). Transport of lipids from Golgi to plasma membrane is defective in Tangier disease patients and Abc1-deficient mice. Nat. Genet..

[74] Kennedy M.A., Barrera G.C., Nakamura K., Baldán Á., Tarr P., Fishbein M.C., Frank J., Francone O.L., Edwards P.A. (2005). ABCG1 has a critical role in mediating cholesterol efflux to HDL and preventing cellular lipid accumulation. Cell Metab..

[75] Yu L., Hammer R.E., Li-Hawkins J., von Bergmann K., Lutjohann D., Cohen J.C., Hobbs H.H. (2002). Disruption of Abcg5 and Abcg8 in mice reveals their crucial role in biliary cholesterol secretion. Proc. Natl. Acad. Sci. USA.

[76] Braun A., Trigatti B.L., Post M.J., Sato K., Simons M., Edelberg J.M., Rosenberg R.D., Schrenzel M., Krieger M. (2002). Loss of SR-BI Expression Leads to the Early Onset of Occlusive Atherosclerotic Coronary Artery Disease, Spontaneous Myocardial Infarctions, Severe Cardiac Dysfunction, and Premature Death in Apolipoprotein E–Deficient Mice. Circ. Res..

[77] Lingwood D., Simons K. (2010). Lipid Rafts As a Membrane-Organizing Principle. Science.

[78] Chernomordik L., Chanturiya A., Green J., Zimmerberg J. (1995). The hemifusion intermediate and its conversion to complete fusion: Regulation by membrane composition. Biophys. J..

[79] Rawle R.J., van Lengerich B., Chung M., Bendix P.M., Boxer S.G. (2011). Vesicle Fusion Observed by Content Transfer across a Tethered Lipid Bilayer. Biophys. J..

[80] Akimov S.A., Molotkovsky R.J., Kuzmin P.I., Galimzyanov T.R., Batishchev O.V. (2020). Continuum Models of Membrane Fusion: Evolution of the Theory. Int. J. Mol. Sci..

[81] Mukherjee S., Maxfield F.R. (2000). Role of Membrane Organization and Membrane Domains in Endocytic Lipid Trafficking. Traffic.

[82] Engelman D.M. (2005). Membranes are more mosaic than fluid. Nat. Cell Biol..

[83] Fuller N., Rand R. (2001). The Influence of Lysolipids on the Spontaneous Curvature and Bending Elasticity of Phospholipid Membranes. Biophys. J..

[84] Plochberger B., Sych T., Weber F., Novacek J., Axmann M., Stangl H., Sezgin E. (2020). Lipoprotein Particles Interact with Membranes and Transfer Their Cargo without Receptors. Biochemistry.

[85] Axmann M., Sezgin E., Karner A., Novacek J., Brodesser M.D., Röhrl C., Preiner J., Stangl H., Plochberger B. (2019). Receptor-Independent Transfer of Low Density Lipoprotein Cargo to Biomembranes. Nano Lett..

[86] Plochberger B., Röhrl C., Preiner J., Rankl C., Brameshuber M., Madl J., Bittman R., Ros R., Sezgin E., Eggeling C. (2017). HDL particles incorporate into lipid bilayers—A combined AFM and single molecule fluorescence microscopy study. Sci. Rep..

[87] Waldie S., Sebastiani F., Browning K., Maric S., Lind T.K., Yepuri N., Darwish T.A., Moulin M., Strohmeier G., Pichler H. (2020). Lipoprotein ability to exchange and remove lipids from model membranes as a function of fatty acid saturation and presence of cholesterol. Biochim. Biophys. Acta Mol. Cell Biol. Lipids.

[88] Maric S., Lind T.K., Raida M.R., Bengtsson E., Fredrikson G.N., Rogers S., Moulin M., Haertlein M., Forsyth V.T., Wenk M.R. (2019). Time-resolved small-angle neutron scattering as a probe for the dynamics of lipid exchange between human lipoproteins and naturally derived membranes. Sci. Rep..

[89] Bruno M.J., Koeppe E.K., Andersen O.S. (2007). Docosahexaenoic acid alters bilayer elastic properties. Proc. Natl. Acad. Sci. USA.

[90] Kahn E., Baarine M., Dauphin A., Ragot K., Tissot N., Seguin A., Ménétrier F., Kattan Z., Bachelet C.-M., Frouin F. (2011). Impact of 7-ketocholesterol and very long chain fatty acids on oligodendrocyte lipid membrane organization: Evaluation via LAURDAN and FAMIS spectral image analysis. Cytom. Part A.

[91] Robins S.J., Fasulo J.M. (1999). Delineation of a novel hepatic route for the selective transfer of unesterified sterols from high-density lipoproteins to bile: Studies using the perfused rat liver. Hepatology.

[92] Wüstner D. (2005). Mathematical Analysis of Hepatic High Density Lipoprotein Transport Based on Quantitative Imaging Data. J. Biol. Chem..

[93] Wüstner D., Mondal M., Huang A., Maxfield F. (2004). Different transport routes for high density lipoprotein and its associated free sterol in polarized hepatic cells. J. Lipid Res..

[94] Robins S.J., Fasulo J.M., Leduc R., Patton G.M. (1989). The transport of lipoprotein cholesterol into bile: A reassessment of kinetic studies in the experimental animal. Biochim. Biophys. Acta.

[95] Bravo E., Botham K., Mindham M.A., Mayes P.A., Marinelli T., Cantafora A. (1994). Evaluation in vivo of the differential uptake and processing of high-density lipoprotein unesterified cholesterol and cholesteryl ester in the rat. Biochim. Biophys. Acta.

